# A Systematic Review of the Applications of Deep Learning for the Interpretation of Positron Emission Tomography Images of Patients with Lymphoma

**DOI:** 10.3390/cancers17010069

**Published:** 2024-12-29

**Authors:** Theofilos Kanavos, Effrosyni Birbas, Theodoros P. Zanos

**Affiliations:** Institute of Health System Science, Feinstein Institutes for Medical Research, Manhasset, NY 11030, USA; tkanavos@northwell.edu (T.K.); ebirbas@northwell.edu (E.B.)

**Keywords:** lymphoma, DLBCL, positron emission tomography, PET, artificial intelligence, AI, machine learning, deep learning, convolutional neural network, CNN

## Abstract

**Background**: Positron emission tomography (PET) is a valuable tool for the assessment of lymphoma, while artificial intelligence (AI) holds promise as a reliable resource for the analysis of medical images. In this context, we systematically reviewed the applications of deep learning (DL) for the interpretation of lymphoma PET images. **Methods**: We searched PubMed until 11 September 2024 for studies developing DL models for the evaluation of PET images of patients with lymphoma. The risk of bias and applicability concerns were assessed using the prediction model risk of bias assessment tool (PROBAST). The articles included were categorized and presented based on the task performed by the proposed models. Our study was registered with the international prospective register of systematic reviews, PROSPERO, as CRD42024600026. **Results**: From 71 papers initially retrieved, 21 studies with a total of 9402 participants were ultimately included in our review. The proposed models achieved a promising performance in diverse medical tasks, namely, the detection and histological classification of lesions, the differential diagnosis of lymphoma from other conditions, the quantification of metabolic tumor volume, and the prediction of treatment response and survival with areas under the curve, F1-scores, and *R*^2^ values of up to 0.963, 87.49%, and 0.94, respectively. **Discussion**: The primary limitations of several studies were the small number of participants and the absence of external validation. In conclusion, the interpretation of lymphoma PET images can reliably be aided by DL models, which are not designed to replace physicians but to assist them in managing large volumes of scans through rapid and accurate calculations, alleviate their workload, and provide them with decision support tools for precise care and improved outcomes.

## Simple Summary

Positron emission tomography (PET) is useful for assessing lymphomas, while artificial intelligence (AI) shows potential as a reliable resource for analyzing medical images. In this context, several studies have developed AI models for the automated interpretation of lymphoma PET images. The proposed models achieved promising performance in various medical tasks, including detection, histological subtyping, differential diagnosis, and prognostication. AI techniques for lymphoma PET evaluation are not designed to replace physicians but to assist them in handling large volumes of scans through rapid and accurate calculations, reduce their workload, and provide them with decision support tools that can improve their ability to deliver precise care for better outcomes.

## 1. Introduction

Lymphomas represent a heterogeneous group of hematological malignancies derived from lymphocytes and are estimated to account for approximately 5% of all cancers. They are broadly classified into Hodgkin lymphoma (HL) and non-Hodgkin lymphoma (NHL), which constitute about 10% and 90% of all cases, respectively. Each category is further divided into several subtypes (Table 1) [1]. HL is rare and considered one of the most highly curable malignancies in both pediatric and adult patients with a survival rate that exceeds 90% [2,3]. NHLs are categorized into aggressive and indolent forms based on their rate of progression and this distinction has substantial therapeutic and prognostic implications. Aggressive types grow and spread rapidly, while indolent ones generally progress slowly [4]. Diffuse large B cell lymphoma (DLBCL) is the most common aggressive NHL, accounting for 80% of all cases [5], whereas follicular lymphoma (FL) is the most frequent indolent NHL, representing 70% of all cases [6].

Positron emission tomography (PET) is a sophisticated physiology-based imaging modality that provides information regarding the function of tissues. The technique relies on detecting radioactivity emitted after the intravenous injection of a radiotracer. The most widely used radioactive tracer is ^18^F-fluorodeoxyglucose (^18^F-FDG), which is a radiolabeled glucose analog and therefore an imaging biomarker for glucose metabolism. Consequently, ^18^F-FDG PET enables the visualization of glucose consumption by tissues and can be used to detect metabolically active lesions [7,8]. In addition, PET can be combined with other cross-sectional imaging modalities, namely, computed tomography (CT) or magnetic resonance imaging (MRI), to provide a comprehensive view that integrates both functional and anatomical information. In other words, PET/CT and PET/MRI provide physicians with physiological details linked to a specific anatomical site, which enhances the accuracy of disease characterization [9]. Although PET comes with its own set of limitations, including radiation exposure, false positive and negative results, cost and availability issues, and interpretation variability, it constitutes a powerful imaging tool for the staging, treatment response evaluation, and post-treatment surveillance in patients with either HL or NHL [10].

Artificial intelligence (AI) refers to the broad concept of computers performing tasks that would typically require human intelligence, such as decision-making, pattern recognition, and problem-solving. Machine learning (ML) is a subfield of AI that involves the development of models that can learn from data without being explicitly programmed to make predictions or identify patterns. Deep learning (DL) is a specific type of ML that uses artificial neural networks to analyze large volumes of complex data [11]. Convolutional neural networks (CNNs) constitute a DL architecture that is particularly effective in processing and analyzing image data [12]. In recent years, AI has emerged as a revolutionary technology in the medical field due to the need to enhance diagnostic accuracy and streamline clinical workflows. A large number of DL models have been developed by multiple scientific groups for the analysis of various kinds of histopathological [13], cytopathological [14], radiological [15], and nuclear medicine [16] images.

**Figure 1:**
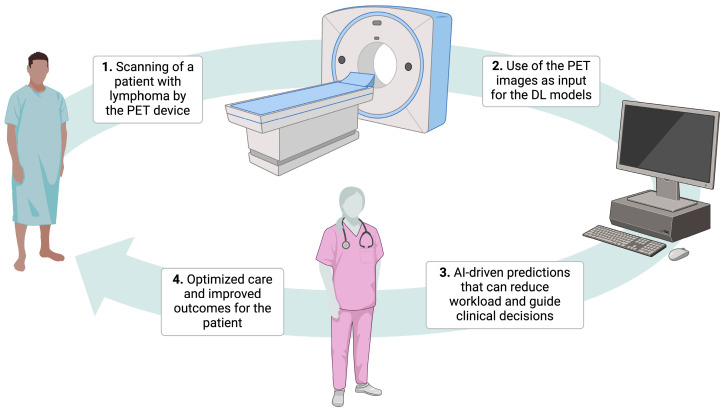
The role of artificial intelligence in optimizing the management of patients with lymphoma using positron emission tomography images without replacing the human touch. Abbreviations: AI, artificial intelligence; DL, deep learning; PET, positron emission tomography. Created using BioRender. Kanavos, T. (2024) https://BioRender.com/w36u293, accessed on 23 December 2024.

As far as lymphomas are concerned, AI-powered models are continuously being developed with the objective of assisting physicians in the management of these hematological malignancies and optimizing clinical workflows. Many of these models are based on PET, which serves as a powerful tool for various types of lymphoma (Figure 1).

**Figure 2:**
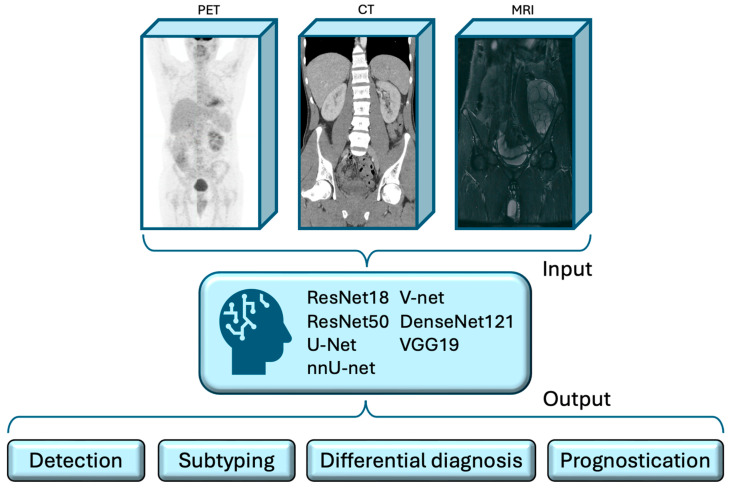
Artificial intelligence-assisted workflow for the analysis of lymphoma medical images. Deep learning models can use positron emission tomography images with or without computed tomography or magnetic resonance imaging scans as inputs to deliver valuable clinical outputs. Abbreviations: CT, computed tomography; MRI, magnetic resonance imaging; PET, positron emission tomography.

In this context, the present systematic review aims to outline the applications of DL for the interpretation of PET images of patients with lymphoma and highlight the potential of AI models to support physicians without replacing their clinical expertise. To provide a comprehensive overview of this topic, we structured our manuscript by categorizing relevant studies based on the distinct medical tasks that AI can facilitate (Figure 2).

## 2. Materials and Methods

Our systematic review was performed in accordance with the recommendations of the preferred reporting items for systematic reviews and meta-analyses (PRISMA) guidance [17]. In addition, the present study was registered with the international prospective register of systematic reviews, PROSPERO, as CRD42024600026.

### 2.1. Search Strategy

We performed a systematic search of PubMed from its inception to 11 September 2024 to identify primary studies that involved the development of a DL model for the interpretation of PET images of patients with lymphoma. For this purpose, the following search algorithm was applied: (deep learning OR convolutional neural network OR CNN) AND (PET OR positron) AND lymphoma.

### 2.2. Eligibility Criteria

Eligible studies were selected based on the following inclusion and exclusion criteria. To be eligible for inclusion, studies needed to focus on the development of at least one DL model for the interpretation of PET images of patients diagnosed with lymphoma. The exclusion criteria encompassed reviews, case reports, datasets, editorials, comments, letters, interviews, preprints, articles published in languages other than English, papers involving non-human subjects, and studies that solely addressed lesion segmentation.

### 2.3. Selection Process

The titles and abstracts of all the citations collected using the previously described methodology were independently screened by two authors using the Rayyan web application [18]. During this screening period, the researchers convened regularly to discuss any disagreements and resolve conflicts by consensus. The full texts of potentially eligible articles identified through this process were then retrieved for further examination to determine the articles that were ultimately included in our systematic review.

### 2.4. Data Extraction Process

To streamline the data extraction process, we generated a spreadsheet form that all authors could access to import data from the included articles. From each study, we manually extracted information regarding the authors, the year of publication, the number of participants and their mean or median age, the imaging modality, the medical objective, the DL architecture, the performance metrics, and whether external validation was conducted.

### 2.5. Study Quality Assessment

The risk of bias and applicability concerns of the studies included in our systematic review were assessed using the prediction model risk of bias assessment tool (PROBAST), which is designed for studies aiming to develop, validate, or update prediction models that can be either diagnostic or prognostic. To accommodate the specific needs of AI-based models, the signaling questions within the tool were adjusted accordingly. PROBAST is organized into four domains, namely, participants, predictors, outcomes, and analysis. The risk of bias was evaluated across all four domains and was rated as low, unclear, or high for each of them. Concerns regarding applicability were assessed for the first three of the aforementioned domains and were judged similarly to the risk of bias. This evaluation was performed by two independent authors and disagreements were resolved through discussion and consensus. An overall judgment about the risk of bias and applicability concerns was also established based on the classifications for each domain. A model with a low risk of bias in all domains was considered to present a low overall risk of bias. The presence of an unclear risk of bias in at least one domain in the absence of a high risk of bias in any domain rendered the overall risk of bias unclear. A high overall risk of bias was assigned if a high risk of bias was noted in any domain. The judgment on the overall applicability concerns was governed by the same rules [19,20].

## 3. Results

This section provides a comprehensive overview of the papers that are relevant to the topic of our systematic review, detailing the selection, characteristics, risk of bias, and key findings of the included studies.

### 3.1. Study Selection

**Figure 3:**
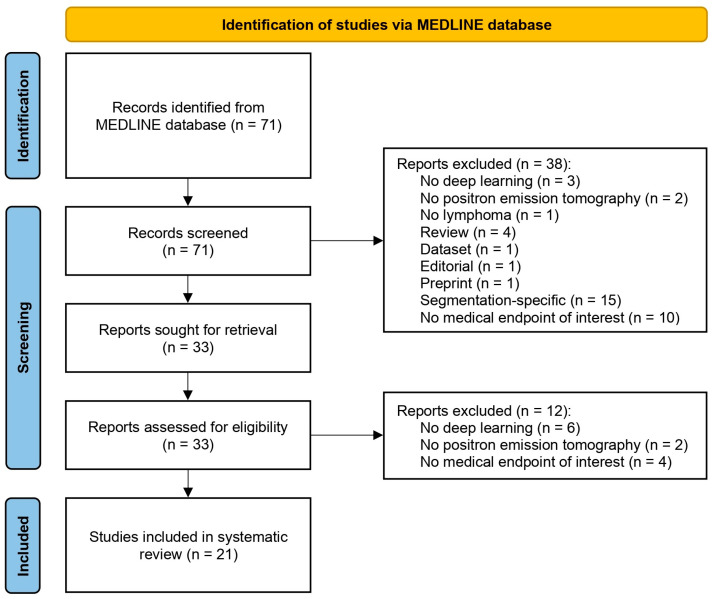
Flow diagram presenting the study selection process along with the number of included and excluded papers.

Our systematic search yielded 71 articles, 38 of which were excluded during the title and abstract screening phase. As a result, 33 papers were selected for full-text assessment. In the end, 21 articles were deemed to fulfill our criteria of eligibility and were included in our review (Figure 3).

### 3.2. Study Characteristics

**Figure 4:**
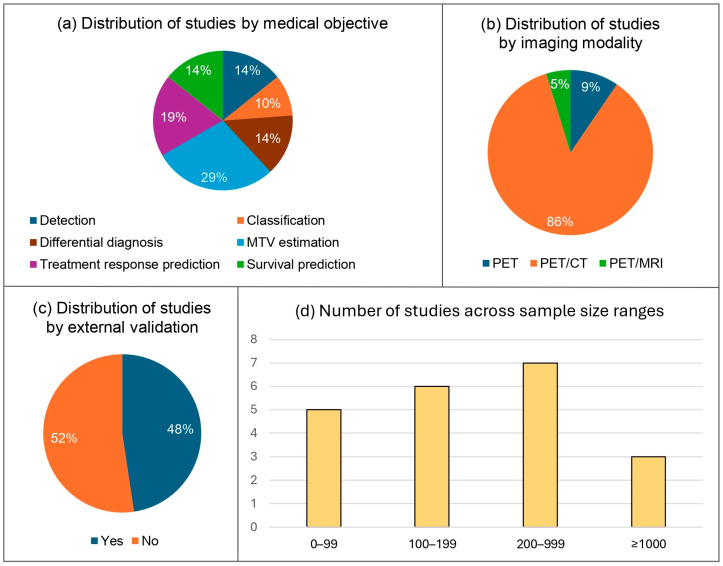
Graphs illustrating the proportion of studies focusing on each different medical task (**a**), the proportion of studies that used each imaging modality (**b**), the proportion of studies that conducted external validation versus those that did not (**c**), and the distribution of studies by sample size (**d**). Abbreviations: CT, computed tomography; MRI, magnetic resonance imaging; MTV, metabolic tumor volume; PET, positron emission tomography.

The articles included in our systematic review were diverse in terms of their medical objectives and methodologies (Figure 4). The number of participants in the studies varied from 39 to 2030 with a median of 167. Sixteen papers (76%) included at least 100 individuals, while three (14%) considered 1000 or more participants. With respect to imaging modalities, 18 articles (86%) were based on PET/CT, two (9%) on PET alone, and only one (5%) on PET/MRI. In terms of medical tasks, three papers (14%) focused on detection, two (10%) on classification, three (14%) on differential diagnosis, six (29%) on metabolic tumor volume (MTV) estimation, four (19%) on treatment response prediction, and three (14%) on survival prediction. Regarding lymphoma types, nine studies (43%) investigated DLBCL, one (5%) focused on mantle cell lymphoma (MCL), one (5%) utilized participants diagnosed with extranodal NK/T cell lymphoma, nasal type (ENKTL-NT), one (5%) involved patients with HL, four (19%) used different types of NHL, three (14%) included both HL and NHL cases, while two papers (9%) did not specify the histological subtypes addressed. All 21 studies (100%) utilized retrospective data, three (14%) used data from open-access sources, two (10%) examined exclusively pediatric cases, three (14%) reported excluding images of poor quality, and 10 (48%) performed external validation.

### 3.3. Risk of Bias in Studies

**Figure 5:**
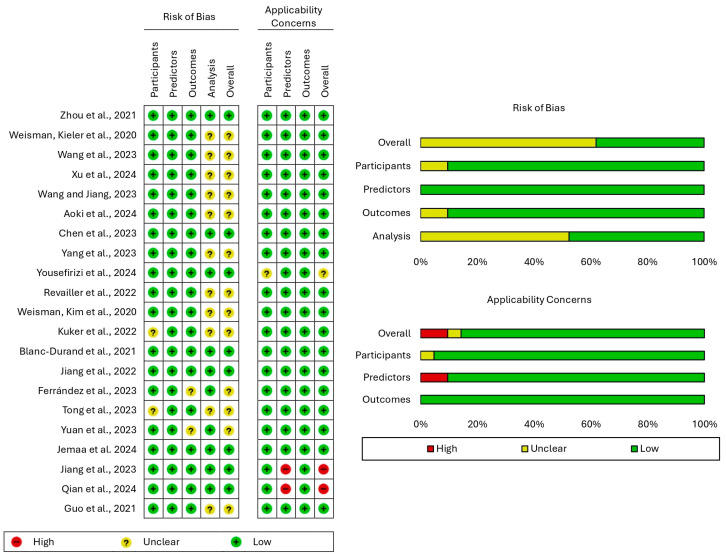
Results of the evaluation of the risk of bias and concerns regarding applicability using the prediction model risk of bias assessment tool (PROBAST).

The risk of bias and concerns regarding the applicability of the included studies were evaluated using PROBAST. Although none of the studies were classified as having a high overall risk of bias, approximately 62% of them were found to have an unclear one. In particular, the small sample size and the lack of external validation were the primary limitations of multiple studies. With respect to applicability, the majority of studies presented low overall concerns. However, one study [21] raised unclear concerns in the domain of participants due to the additional use of lung cancer and melanoma scans for model training, while two studies [22,23] exhibited high concerns in the domain of predictors because they considered clinical parameters alongside PET/CT scans (Figure 5).

### 3.4. Results of Included Studies

Below, we present the findings of the included studies, organized by their primary objective, namely, detection, classification, differential diagnosis, MTV estimation, and treatment response and survival prediction (Table 2).

#### 3.4.1. Detection

Some research groups have proposed DL models for the automatic detection of lymphoma lesions in PET images, which can rapidly provide clinicians with useful information about the location and burden of a neoplasm. The manual identification of multiple lymphoma lesions in medical images can be a tedious and lengthy process; therefore, models that automatically perform this task could significantly ease the workload for physicians and potentially limit the time interval between the acquisition and interpretation of PET images.

The quantitative analysis of PET/CT images of patients with lymphoma offers significant prognostic insights but requires the identification of all lesions, which can be time-consuming, labor-intensive, and susceptible to inter-reader variability. To address this issue, Zhou et al. [24] constructed an Xception-based U-Net for the computer-aided detection of MCL in PET/CT images. When tested on external institution scans, the model achieved a median sensitivity of 84% with 14 false positives (FPs) per patient. Interestingly, the performance of the model did not exhibit a statistically significant difference between the images from within and outside the institution, which indicates its robustness and applicability to independent datasets. It is worth mentioning that most FPs were limited to sites where normal physiological processes or benign inflammatory conditions could explain the findings. In addition, the model demonstrated relatively low sensitivity for lesions with a maximum standardized uptake value (SUV_max_) beneath the liver reference, which may correspond to indolent MCL cases. Similarly, Weisman et al. [25] developed an ensemble of three-dimensional CNNs for the automatic detection of lymph nodes involved in lymphoma in PET/CT images, which achieved a sensitivity of 85% with an average of four FPs per patient and performed comparably to the differences observed between the annotations of two physicians. Furthermore, Wang et al. [26] generated a multimodal DL algorithm for the computer-aided identification of lymphoma lesions in PET/MRI scans of pediatric patients, which performed adequately with a sensitivity of 76% and a false discovery rate of 10%.

#### 3.4.2. Classification

As already mentioned, lymphomas are classified into HL and NHL and each category is further divided into multiple subtypes, the determination of which has both therapeutic and prognostic implications. A biopsy followed by a histopathological examination is necessary for the definite diagnosis and subtype identification of lymphomas; however, such an approach comes with certain drawbacks. Needle core biopsy may not always provide sufficient information for an accurate analysis due to the spatial and temporal heterogeneity of lymphomas, while excision biopsy carries the risk of anesthetic and surgical complications. On top of that, the histopathological examination of lymphoma samples under the microscope is a time-consuming and challenging task [4,27].

A study involving both aggressive and indolent NHL cases indicated that ^18^F-FDG uptake is higher in aggressive than in indolent forms and that an SUV_max_ greater than 10 may predict aggressive behavior [41]. However, in another study of 38 indolent subtypes of NHL, the SUV_max_ of lymphomas widely ranged from 1.7 to 41.2, suggesting that the SUV_max_ can vary significantly across different histological subtypes of indolent NHL [42]. Consequently, adopting a single SUV_max_ cut-off for distinguishing between aggressive and indolent NHLs on PET/CT images would be unreliable. Therefore, although nuclear medicine experts can identify lymphomas on PET/CT images based on abnormal ^18^F-FDG avidity and anatomical features, such as lymph node enlargement, they cannot reliably determine the specific lymphoma subtype solely based on imaging findings [4].

Given the limitations of visual inspection of medical images for lymphoma classification and the challenges associated with histopathological examination, the implementation of AI models that predict lymphoma subtypes based on PET/CT scans in clinical practice would be highly beneficial for physicians involved in the management of patients with lymphoma. Such models have been recently proposed by two research groups.

Xu et al. [4] developed a hybrid few-shot multiple-instance learning model to distinguish between FL and DLBCL using PET/CT images from 61 NHL patients. The model achieved an area under the curve (AUC) of 0.795 and outperformed its traditional counterparts, which used radiomic features, random forests, and support vector machines (SVMs). Their approach utilized hybrid features generated by fusing DL-based and radiomic features, which enhanced classification precision. Combining hybrid features with self-supervised learning, few-shot learning, and multiple-instance learning efficiently addresses challenges related to data scarcity and incomplete annotations.

A more thorough NHL classification was sought by Wang and Jiang [27], who built a light-weighted pseudo-spatial-temporal radiomic network for the classification of NHL into eight different subtypes, namely, DLBCL, anaplastic large cell lymphoma, Burkitt lymphoma (BL), NK/T cell lymphoma, FL, MCL, peripheral T cell lymphoma, and lymphoblastic lymphoma, using PET/CT images from 80 patients. The model employed a novel radiomics method that extracted both global and local radiomic features from lymphoma PET/CT images to enhance intra-tumor heterogeneity classification and thus enable accurate NHL subtyping. This method outperformed its competing ones, achieving an AUC of 0.902.

#### 3.4.3. Differential Diagnosis

The differential diagnosis of lymphomas is extensive as numerous diseases can mimic the clinical and radiological appearance of these malignancies. Given that lymphomas require very specific therapeutic approaches, rapid and accurate differentiation from other diseases resembling them is paramount. This ensures the timely initiation of the indicated therapeutic plan, prevents unnecessary and inappropriate treatments, and ultimately improves the patient’s outcome.

Differentiation between sarcoidosis and DLBCL is crucial as sarcoidosis is a benign systematic disease managed by a watch-and-wait approach or immunosuppressive therapy, whereas DLBCL exhibits an aggressive behavior and generally requires prompt treatment with immunochemotherapy [43,44]. While histopathological examination is the gold standard method for differentiating between the two entities, obtaining biopsies from lesions located in deep anatomical structures or near blood vessels can be especially difficult [28]. To address the above challenges, Aoki et al. [28] proposed a CNN model to distinguish sarcoidosis from DLBCL using PET/CT images. The model achieved a high level of performance and differentiated sarcoidosis from DLBCL based on differences in the distribution of ^18^F-FDG accumulation with an AUC of 0.963.

Primary breast lymphoma is a rare malignancy, accounting for 0.4% of all breast cancers, and DLBCL is recognized as its most common histological subtype [45]. Distinguishing breast lymphoma from the far more prevalent breast carcinoma is challenging as physical examination and standard imaging modalities, namely, mammography, ultrasonography (US), CT, and MRI, reveal overlapping characteristics [46,47]. Therefore, the diagnosis of breast lymphoma is usually feasible only after a breast biopsy. However, needle biopsy can lead to misdiagnosis, especially if necrotic tissue is sampled when puncturing large breast nodules containing necrotic regions. Furthermore, breast lymphoma is primarily managed with chemotherapy and radiation in contrast to breast carcinoma, which is mainly treated with surgery [48]. Nonetheless, due to the rarity of breast lymphoma and its clinical and radiological similarities with carcinoma, many lymphomatous nodules are initially misdiagnosed as carcinomas and surgically removed [29,48]. Considering the above, exploring non-invasive techniques to precisely distinguish breast lymphoma from carcinoma is clinically important. In this context, Chen et al. [29] designed a novel attention-based aggregate CNN model for the non-invasive and accurate differentiation of breast DLBCL from invasive ductal carcinoma using PET/CT images from 236 patients. The AUC of this model showed an improvement of nearly 5% compared to previous models, reaching 0.886 in an internal and 0.788 in an external dataset.

A significant differential diagnostic challenge frequently encountered in clinical practice is the distinction between metastatic and lymphomatous cervical lymphadenopathy. This difficulty arises from the overlapping clinical and radiological features of the two entities. While US-guided fine needle aspiration cytology (FNAC) is a valuable tool for lymphadenopathy investigation, it presents certain limitations that inhibit timely and precise lymphoma diagnosis. More specifically, the adequacy of the sample can vary significantly depending on the physician’s expertise and the specific location of the suspicious lymph node. Additionally, FNAC may lead to misdiagnosis as it may fail to capture the entire tumor architecture, while there is a risk of misinterpreting lymphomas, especially low-grade NHLs with minor cytomorphological atypia, as reactive lymphoid hyperplasia and vice versa [30,49]. In response to these difficulties, Yang et al. [30] developed a ResNet50-based model to distinguish between lymph node metastases from solid tumors and lymphoma involvement in enlarged cervical lymph nodes using PET/CT scans. Their model integrated both handcrafted and DL-based radiomic features and achieved a remarkable performance, reaching an AUC of 0.948. This is the first study to use DL to distinguish metastatic from lymphomatous enlarged cervical lymph nodes, paving the way for further research in this direction.

#### 3.4.4. Metabolic Tumor Volume Estimation

MTV is a measure of the metabolically active volume of tumors derived from ^18^F-FDG PET. It is calculated as the sum of the volume of voxels with a standardized uptake value (SUV) that exceeds a particular threshold within the tumor [50]. MTV has demonstrated substantial prognostic value in both HL [51] and various types of NHL, including DLBCL [52], FL [53], MCL [54], BL [55], primary mediastinal large B cell lymphoma [56], peripheral T cell lymphoma [57], and T cell lymphoblastic lymphoma [58]. The quantification of MTV through the manual delineation of multiple lesions throughout the body constitutes a time-consuming and operator-dependent process. To address this issue, several studies have focused on developing automated methods for the rapid, reliable, and consistent computation of MTV of lymphomas as part of daily workflow.

A couple of research groups have studied various lymphoma types. To facilitate automated MTV calculation from lymphoma PET/CT images, Yousefirizi et al. [21] constructed a reliable two-step cascaded segmentation approach, the predictions of which demonstrated a strong correlation with ground truth values with an *R*^2^ of 0.89. Similarly, another research group developed an automated method for the quantification of MTV from PET/CT images of patients with ^18^F-FDG-avid lymphomas, namely, DLBCL, FL, and HL. The model performed impressively with a Spearman’s correlation coefficient (SCC) between the manually extracted and predicted MTV reaching 0.98 [31].

Other studies have focused on specific types of lymphoma. One of them proposed a three-dimensional patch-based, multi-resolution pathway CNN architecture for the extraction of MTV from baseline PET/CT images of pediatric HL cases. The study demonstrated strong agreement between the automated and physician-determined MTV with a Pearson’s correlation coefficient (PCC) of 0.88 although the former was slightly underestimated [32]. Furthermore, Kuker et al. [33] developed a DL model to automatize the estimation of MTV from PET/CT images of DLBCL and reported high concordance in calculations between the automated method and two nuclear medicine physicians with PCCs and intraclass correlation coefficients (ICCs) around 0.98. The same task was also addressed by another research group, which reported that, although their model tended to slightly underestimate MTV, the predicted values showed a strong correlation with the ground truth measurements, achieving an *R*^2^ of 0.82 in an external validation cohort [34]. Finally, Jiang et al. [35] constructed a three-dimensional U-Net architecture to quantify MTV in DLBCL using only PET images. They reported a notable positive correlation between the ground truth and computer-derived MTV with an *R*^2^ of 0.94 in an external validation cohort. In addition, the predicted MTV was proven to be an independent prognostic factor of progression-free survival (PFS) and overall survival (OS) in DLBCL patients.

#### 3.4.5. Treatment Response Prediction

Treatment response prediction can be very helpful to healthcare providers involved in the management of patients with lymphoma as it can guide therapeutic decisions and personalize treatment plans. Recent advancements in AI have led to a growing interest in utilizing DL models to evaluate treatment outcomes based on PET images.

Ferrández et al. [36] investigated the capability of a CNN to predict the treatment outcomes of patients with DLBCL using maximum intensity projection (MIP) images from baseline PET/CT scans. Their model was designed to predict whether a patient would experience a short time to progression (TTP) of up to two years or not and achieved an AUC of 0.74 in an external dataset, demonstrating superior performance compared to an international prognostic index (IPI)-based model. In a subsequent study, the authors aimed to validate their method in five external datasets from different clinical trials and showed that their model remained predictive of treatment outcome and outperformed the IPI score in all five independent clinical trials [59]. Similarly, another scientific group used a DL-based method on pre-treatment PET images to predict the response of DLBCL and FL to chimeric antigen receptor (CAR) T cell therapy. The average AUC of the lesion-level response prediction using single whole slice-based input was 0.93. Additionally, the model could adequately predict patient-level treatment response using a rule-based reasoning approach applied to the lesion-level prediction results. This methodology holds promise for providing valuable prognostic insights for clinical decision-making prior to the initiation of CAR T cell therapy [37].

Yuan et al. [38] developed a multimodal DL model to predict primary treatment failure in DLBCL using interim PET/CT images. Their hybrid learning model was optimized with a contrastive training objective and achieved an AUC of 0.925 in an external dataset. The ability of the model to accurately predict primary treatment failure in patients with DLBCL receiving the front-line standard of care suggests that it has the potential to guide personalized treatment decisions. Furthermore, another study proposed a DL-based algorithm for assessing treatment response in NHL according to the Lugano 2014 classification using PET/CT scans. The Lugano 2014 classification is currently the standard method for the assessment of treatment response in NHL. It considers four categories of treatment response based on PET/CT images, namely, complete metabolic response (CMR), partial metabolic response, no metabolic response, and progressive metabolic disease. Obtaining a CMR by the end of treatment offers prognostic value. Hence, the authors evaluated the performance of the model in distinguishing between CMR and non-CMR cases and reported sensitivities and specificities ranging from 0.85 to 0.97 and from 0.50 to 0.78, respectively, across different test datasets [39].

#### 3.4.6. Survival Prediction

The survival of patients with lymphoma varies significantly among individuals [60]. The integration of radiomics and AI into prognosis prediction tools aims to achieve more accurate prognostic evaluation and improve clinical decision-making for patients with lymphoma [61].

Identifying DLBCL patients at increased risk of relapse who would benefit from intensified chemotherapy is critical, given that 30–40% of DLBCL patients experience disease recurrence and die [22,62]. Although widely used in clinical practice, the IPI has been found to be insufficient to precisely identify DLBCL patients with an unfavorable prognosis [22,63]. In a retrospective study of 684 DLBCL patients conducted by Jiang et al. [22], DL scores, namely, DL-based imaging biomarkers, were extracted from PET/CT images and integrated along with clinical risk factors and metabolic metrics into a multiparametric model for prognosis prediction. This model outperformed the IPI and a conventional model that only comprised metabolic metrics and clinical variables, achieving C-indices for PFS and OS of 0.760 and 0.770, respectively, in an external validation cohort. Similarly, Qian et al. [23] developed another multiparametric model that combined DL-based radiomic features with clinical and PET parameters for prognosis prediction in DLBCL patients. The model achieved C-indices for PFS and OS of 0.770 and 0.771, respectively, in an external validation dataset. The above studies indicate that DL-based radiomic features could operate as reliable imaging biomarkers for precise survival prediction in DLBCL patients and that multiparametric models incorporating these features could enable personalized prognosis assessment and customized treatment strategies for DLBCL patients [22,23].

ENKTL-NT is an uncommon type of lymphoma with a poor prognosis [64,65]. This aggressive neoplasm is strongly associated with Epstein-Barr virus (EBV) and is more frequent in Asia and Central and South America, where it accounts for 5–15% of all lymphomas, compared to the United States and Europe, where it represents only 0.2–0.4% of NHLs [66]. Due to its rarity and the resulting limited data availability, predicting the prognosis of ENKTL-NT is challenging. Although studies have shown that some PET metrics, such as SUV_max_, mean SUV (SUV_mean_), MTV, and total lesion glycolysis (TLG), are correlated with survival, these findings are considered controversial [40]. To overcome the issue of data scarcity, Guo et al. [40] developed a weakly supervised DL model for the prognosis prediction of ENKTL-NT using PET/CT scans from 167 patients. The proposed method successfully included 83 patients with missing or incomplete follow-up data together with 64 patients with complete follow-up data in the training process. The model achieved an AUC of 0.875 for the prediction of PFS and outperformed a conventional model trained solely on patients with complete follow-up data.

To further enhance survival prediction and risk stratification in patients with lymphoma, recent studies have explored novel metrics that reflect tumor dissemination and are derived from baseline PET/CT scans. These include the maximum distance between two lesions (Dmax) as well as the largest distance between the spleen and a lesion (Dspleen) [67,68]. It has been shown that high Dmax normalized with the body surface area and MTV constitute independent prognostic factors in DLBCL, which complement each other in characterizing the tumor by capturing two different aspects of the disease, namely, its spread and burden, respectively [67]. Moreover, a study has demonstrated that Dspleen normalized with the body surface area strongly predicts survival in DLBCL and its integration with MTV and IPI can further improve prognostication [68]. The quantification of the above PET-based biomarkers can be assisted by DL tools, bridging the gap between AI and clinical workflows. Indeed, Girum et al. [69] developed a DL model for the automated estimation of surrogate Dmax from only two MIP images of whole-body PET scans in patients with DLBCL. The AI-derived surrogate Dmax was highly correlated with its original counterpart and was effective in predicting PFS and OS, offering a streamlined approach to prognostication in DLBCL patients [69].

## 4. Discussion

Over the past decade, the implementation of AI in healthcare and particularly in medical imaging has increased. As a result, a growing number of DL models are continuously being developed for diverse diagnostic and prognostic tasks, many of which are based on imaging data and concern malignant diseases. A substantial effort has been made by several scientific groups to summarize the findings of studies that propose such methodologies in order to inform future research and guide clinical applications. For instance, a recent systematic review focused on DL models for the diagnosis of lung cancer from CT images [70], while another one investigated AI models for the identification of ovarian cancer in US, MRI, and CT scans [71]. To the best of our knowledge, this is the first systematic review to specifically address the performance and feasibility of DL-based methods for the computer-aided analysis of lymphoma PET images.

### 4.1. General Interpretation of the Results

The constant effort of numerous research groups to design DL methods for the computer-aided interpretation of PET scans of individuals with lymphoma represents a major advancement in medical imaging with significant potential to benefit the management of patients. These models showcase promising results across several medical tasks and can therefore assist medical professionals through diverse applications. DL methods for the automatic detection of lymphoma in PET images can ease clinicians’ workload and expedite the identification of multiple lesions with sensitivities ranging from 76% to 85% and FPs per patient between four and 14 [24,25,26]. DL models can also aid in the histological classification of lymphoma using PET scans with AUCs of 0.795 and 0.902 for binary and 8-class subtyping tasks, respectively [4,27]. In addition, PET-based DL algorithms can be helpful in distinguishing lymphomas from other pathological conditions, such as sarcoidosis, breast carcinoma, and lymph node metastases from solid tumors, with AUCs ranging from 0.788 to 0.963 depending on the specific differential diagnosis task [28,29,30]. Furthermore, DL models utilizing PET images can reliably quantify lymphoma MTV, a process that is time-consuming and operator-dependent when performed manually, achieving strong correlations with ground truth measurements with *R*^2^ values of 0.82–0.94 [21,31,32,33,34,35]. Predictive modeling of treatment outcomes and survival constitutes another area where AI shows considerable promise. DL algorithms based on PET scans can effectively anticipate treatment responses in patients with lymphoma and potentially guide personalized therapeutic approaches, demonstrating AUCs between 0.74 and 0.93 [36,37,38,39]. Additionally, DL methods using PET images can provide valuable prognostic insights regarding the survival of lymphoma patients with C-indices of 0.760–0.770 and 0.770–0.771 for PFS and OS, respectively [22,23,40].

The studies included in our systematic review varied widely in regard to their medical objectives. Almost 43% of them centered on the quantification of disease burden, either by simply detecting lymphomatous lesions [24,25,26] or by calculating MTV [21,31,32,33,34,35]. The constant effort by multiple research groups to develop models for this specific task is not surprising, given that the manual evaluation of tumor burden is a challenging and time-consuming procedure and that tumor burden is considered a substantial prognostic factor not only in lymphomas [72] but also in numerous kinds of solid tumors [73,74]. More specifically, one study presented tumor burden as the most significant prognostic marker in early-stage HL [75]. In addition, the prognostic value of disease burden has been demonstrated in several types of NHL, including DLBCL [76], FL [77], and BL [78]. Based on the above, the development of automated methods for the precise and reliable quantification of tumor burden in lymphomas is clinically relevant and could facilitate treatment planning. Interestingly, a recent study proposed a standardized MTV measurement workflow for lymphoma that allowed for highly reproducible MTV quantifications and could act as a reference to test improvements in the calculation of MTV using AI tools [79].

Two of the articles described in our paper considered only pediatric participants [26,32]. Notably, Wang et al. [26] developed a DL algorithm for the automatic detection of lymphoma lesions in PET/MRI scans of pediatric patients. This kind of model is particularly relevant and impactful for two key reasons. Firstly, lymphoma represents the most common type of cancer in adolescents aged 15–19 years and the third most frequent in children up to 14 years old [80]. Secondly, approximately 54–81% of the radiation dose from a whole-body PET/CT scan is attributed to the CT examination, while the remaining amount is the result of the injection of the PET radioactive tracer [81]. The replacement of the CT component with MRI has been reported to reduce the radiation dose by 45–48% [82,83], which is especially important for young patients, who are more sensitive to the potential harmful effects of ionizing radiation [84].

### 4.2. Limitations of the Included Studies

Despite their promising results, the included studies exhibited some limitations. The number of participants across the studies ranged from 39 to 2030 with a median of 167, while roughly one-quarter of the studies included PET images from fewer than 100 patients. The limited dataset size for model development may have affected the performance and predictive ability of the DL models used in these studies [40]. However, there is no consensus regarding the number of patient cases necessary for training and developing a specific DL model, while the dataset size can vary significantly depending on the complexity of the task and the heterogeneity of the training sample [25]. Furthermore, over half of the studies did not conduct external validation, which is essential for assessing a model’s reproducibility and generalizability to diverse and unseen patient populations [85,86]. External validation corroborates a model’s reputation and trustworthiness; therefore, its absence in these studies reduces the potential of their models for clinical implementation [85,86,87]. Another limitation observed in some studies was the need for the manual delineation of lymphoma lesions in medical images during the preprocessing stage, which can be time-consuming and prone to high inter-reader variability [88].

### 4.3. Limitations of the Review

Our systematic review has certain limitations that should be acknowledged. Firstly, our search was confined to a single database, namely, MEDLINE, which was chosen for its extensive coverage of biomedical literature. Additionally, given the rapid pace of advancements in the emerging field that our review focuses on, some relevant articles may have been released after our search was conducted and before our manuscript was published. Furthermore, we focused exclusively on DL models that used images and excluded studies proposing other ML approaches based on different kinds of data that could still provide insights about patients with lymphoma. In addition, we only considered algorithms for the interpretation of lymphoma PET images and did not include studies that presented denoising techniques [89,90] and others that aimed at reducing radiation exposure by generating high-quality diagnostic PET images from ultra-low-dose PET scans [91]. Another limitation of our review is that we excluded studies that solely addressed tumor segmentation in order to concentrate on those dedicated to broader interpretative tasks. Lastly, publication bias could potentially exist since our systematic review was restricted to published literature, which may not represent the entire spectrum of available research. Studies that propose highly accurate models are more likely to be published than others with less attractive results [92].

### 4.4. Implications of the Results for Practice and Future Research

The shortage of clinicians coupled with an increasing patient population has led to an overwhelming workload for healthcare professionals, which can negatively impact their decision-making. Conversely, there is evidence to suggest that a reduction in the clinical workload can improve clinical judgments and therefore positively influence patient outcomes [93]. Excessive workload has also been associated with burnout [94], which in turn negatively affects not only the health of the physicians but also the delivery and cost of care [95]. In this context, the careful integration of AI into healthcare is considered a promising strategy to potentially alleviate the workload of physicians across various clinical activities and thereby mitigate burnout and augment the quality of care [96].

The goal of producing computer-aided methods for medical tasks is not to replace physicians but rather to support them by leveraging the computational power and data processing capabilities of AI [97]. With their ability to perform rapid and precise calculations, DL-based models could assist nuclear medicine physicians, radiologists, and pathologists in managing high volumes of medical images and tissue specimens. In addition, AI algorithms can detect subtle patterns, anomalies, and indicators of disease that may be overlooked by the human eye [98]. Another potential use of AI models is smart worklist prioritization, which could enable the identification of scans that physicians should examine promptly [99]. Ultimately, AI holds the potential to provide physicians with decision support tools to enhance their ability to deliver precise care and reduce their, often overwhelming, workload.

Two principal methodologies are currently used to develop AI-driven imaging biomarkers: radiomics and DL [100]. Radiomics refers to the extraction of quantitative features from medical scans to describe tissue and lesion characteristics, such as shape and intensity [101,102,103]. Radiomics has demonstrated significant potential in cancer screening, diagnosis, prognosis, and treatment response assessment [104]. The predictive value of radiomics can be enhanced through its integration with other types of data. Notably, recent findings indicate that PET/CT radiomics combined with liquid biopsy provides improved outcome prediction in patients with lymphoma compared to imaging alone [105]. Radiomic features can function as inputs for ML models, which in turn can predict various outcomes [100]. In addition to standard radiomics, which relies on static snapshots, delta radiomics emerges as an innovative approach that captures changes in tissues and lesions over time, which has potential in regard to outcome prediction [106,107]. While traditional radiomics methods have proven utility, they are often criticized for introducing human bias and for being prone to reproducibility issues due to the variability in imaging techniques and manual delineation, which is highly observer-dependent [108,109]. In contrast, DL algorithms can automatically extract features directly from raw images without the need for prior manual segmentation [109,110]. Both radiomics and DL are rapidly evolving technologies, which can be combined to revolutionize the field of medical imaging and drive the advancement of precision medicine [111].

The extensive use of PET for lymphoma evaluation has generated an unprecedented volume of imaging data. As a result, there is an urgent need for advanced analytical methods to effectively manage and interpret this data deluge. In this context, DL has emerged as a crucial tool, offering sophisticated methods for analyzing lymphoma PET images that enable timely and precise detection, histological subtyping, differential diagnosis, and prognostication.

While DL presents immense opportunities in regard to lymphoma research, several challenges must be addressed before these models can be deployed in clinical settings. The high heterogeneity of multiple lymphoma subtypes characterized by distinct biological profiles, diverse imaging appearances, and variable clinical outcomes complicates the development of DL models that can effectively generalize across all subtypes [112,113]. Additionally, variations in imaging equipment, scanning protocols, and population characteristics across institutions may restrict the usefulness of AI models trained on data from specific technical, clinical, and socioeconomic settings [114]. Another concern is that AI algorithms often operate as “black boxes” since their decision-making processes might remain obscure and not easily understandable [114,115,116]. This lack of transparency challenges the reliability of DL models as it may be difficult for clinicians to comprehend and trust the rationale behind their predictions, which may hamper the implementation of DL tools in clinical practice [115]. In addition, the opacity of AI methods may hinder the patients’ understanding and thereby affect their ability to provide informed consent and exercise their autonomy [117]. However, when the effectiveness of such models has been sufficiently proven through rigorous validation and robust performance metrics, the cost of providing explanation may exceed the potential benefit [117,118].

To address these limitations, it is vital to utilize large, precisely labeled, and diverse datasets for training and validation, establish standardized imaging protocols, and encourage collaboration among AI developers, clinicians, ethicists, and regulatory bodies. Such efforts are essential to ensure that DL models effectively meet high standards of performance, safety, and fairness across diverse subpopulations while proactively addressing potential biases and ethical concerns [114,119,120]. This holistic approach will facilitate the smooth integration of DL into clinical practice, enhancing physicians’ ability to deliver precise care to lymphoma patients and thereby improve clinical outcomes.

Through our systematic review, we identified several areas where future research regarding the DL-based analysis of lymphoma PET images could focus on. Subsequent studies could further explore models that utilize PET/MRI scans for the evaluation of lymphomas, which are underrepresented in the current literature compared to PET/CT images. Specifically, only one of the 21 articles included in our review was based on PET/MRI. The field would also benefit from the development of innovative methods tailored specifically to pediatric lymphomas, which exhibit distinct epidemiological characteristics, histological distribution, and biological behavior compared to adult cases. Furthermore, DL algorithms for the computer-aided staging of lymphomas using PET images are currently lacking, highlighting a potential direction for future investigation. Finally, the availability of large, high-quality, and accessible datasets of diverse PET images of patients with lymphoma would facilitate the utilization of a wide range of patient populations and lymphoma types, thereby enabling the production of robust models with enhanced generalizability and promoting fairness by ensuring equitable representation across different demographic and clinical subgroups.

## 5. Conclusions

DL models for the analysis of lymphoma PET images have achieved promising performance across different medical tasks, namely, detection, classification, differential diagnosis, MTV calculation, and treatment response and survival prediction. They are not designed to replace physicians but to aid them in handling large volumes of scans, alleviate their workload, and offer them decision support tools that can improve their ability to provide precise care to their patients. Despite the encouraging results of automated methods for analyzing lymphoma PET images, it appears that there is still a gap to be bridged before these models can reach the necessary maturity for widespread use in clinical settings.

## Figures and Tables

**Table 1:** Classification of lymphomas.

Non-Hodgkin Lymphoma	Hodgkin Lymphoma
Aggressive types Diffuse large B cell lymphoma Primary mediastinal large B cell lymphoma Burkitt lymphoma Peripheral T cell lymphoma Anaplastic large cell lymphoma Others	Classical Nodular sclerosis Mixed cellularity Lymphocyte-rich Lymphocyte-depleted
Indolent types Follicular lymphoma Small lymphocytic lymphoma Marginal zone lymphoma Lymphoplasmacytic lymphoma Cutaneous T cell lymphoma Others	Nodular lymphocyte-predominant

**Table 2:** Main characteristics of the studies included in the systematic review.

Study	Number of Participants (Mean/Median Age in Years if Reported)	Imaging Modality	Medical Objective	DL Architecture	External Validation	Performance Metrics
Zhou et al., 2021 [24]	142 (58)	PET/CT	Detection	An Xception-based U-Net	Yes	Se: 84.00% FPs/patient: 14
Weisman, Kieler et al., 2020 [25]	90 (44.47)	PET/CT	Detection	An ensemble of 3D patch-based, multi-resolution pathway CNNs	No	Se: 85.00% FPs/patient: 4
Wang et al., 2023 [26]	73 (16)	PET/MRI	Detection	A multimodal fusion algorithm	No	Se: 76.00% FDR: 10.00%
Xu et al., 2024 [4]	61 (56.49)	PET/CT	Classification	A hybrid few-shot multiple-instance learning model	No	AUC: 0.795 F1-score ^1^: 75.30%
Wang and Jiang, 2023 [27]	80	PET/CT	Classification	A novel model based on SA-R^2^-Net and ResNet	No	AUC: 0.902 F1-score: 85.20%
Aoki et al., 2024 [28]	118 (60.95)	PET/CT	Differential diagnosis	A novel CNN model	No	AUC: 0.963 F1-score: 87.49%
Chen et al., 2023 [29]	236 (51.31)	PET/CT	Differential diagnosis	A novel attention-based aggregate CNN model	Yes	AUC: 0.788 F1-score: 70.90%
Yang et al., 2023 [30]	165	PET/CT	Differential diagnosis	A DL-SVM model based on ResNet50 architecture	No	AUC: 0.948 F1-score: 81.80%
Yousefirizi et al., 2024 [21]	1418	PET/CT	MTV estimation	A cascaded U-Net-based approach	Yes	*R*^2^: 0.89
Revailler et al., 2022 [31]	2030	PET/CT	MTV estimation	A 3D V-Net model	No	SCC: 0.92–0.98
Weisman, Kim et al., 2020 [32]	100 (15.80)	PET/CT	MTV estimation	An ensemble of 3D patch-based, multi-resolution pathway CNNs	No	PCC: 0.88
Kuker et al., 2022 [33]	100	PET/CT	MTV estimation	A model built off of a pre-trained 2D dilated residual U-Net architecture	No	PCC: 0.82 ICC: 0.82
Blanc-Durand et al., 2021 [34]	733	PET/CT	MTV estimation	A 3D U-net architecture with two input channels	Yes	*R*^2^: 0.82
Jiang et al., 2022 [35]	414	PET	MTV estimation	A fully CNN with an nnU-Net architecture	Yes	*R*^2^: 0.94
Ferrández et al., 2023 [36]	636	PET/CT	Treatment response prediction	A dual-branch four-layer CNN	Yes	AUC: 0.74
Tong et al., 2023 [37]	39	PET	Treatment response prediction	Transfer learning using a pre-trained CNN model	No	AUC: 0.93
Yuan et al., 2023 [38]	249	PET/CT	Treatment response prediction	Conv-LSTM-based hybrid learning model optimized with a contrastive training objective	Yes	AUC: 0.925 F1-score: 54.55%
Jemaa et al., 2024 [39]	1418	PET/CT	Treatment response prediction	A novel DL-based algorithm	Yes	Se: 85–97% Sp: 50–78%
Jiang et al., 2023 [22]	684	PET/CT	Survival prediction	A multiparametric model with a DL component based on VGG19 and DenseNet121	Yes	C-index for PFS: 0.760 C-index for OS: 0.770
Qian et al., 2024 [23]	449	PET/CT	Survival prediction	A multiparametric model with a DL component	Yes	C-index for PFS: 0.770 C-index for OS: 0.771
Guo et al., 2021 [40]	167	PET/CT	Survival prediction	A weekly supervised DL model based on ResNet18	No	AUC: 0.875

^1^ F1-score is a machine learning evaluation metric that considers both precision and recall and is calculated as the harmonic mean of these two measures. Abbreviations: 2D, two-dimensional; 3D, three-dimensional; AUC, area under the curve; CNN, convolutional neural network; CT, computed tomography; DL, deep learning; FDR, false discovery rate; FP, false positive; ICC, interclass correlation coefficient; MRI, magnetic resonance imaging; MTV, metabolic tumor volume; OS, overall survival; PCC, Pearson’s correlation coefficient; PET, positron emission tomography; PFS, progression-free survival; SCC, Spearman’s correlation coefficient; Se, sensitivity; Sp, specificity; SVM, support vector machine.
